# Is the continuity of the therapist–patient relationship relevant for the discharge outcome in orthopaedic physical rehabilitation?

**DOI:** 10.1007/s12306-024-00860-y

**Published:** 2024-10-02

**Authors:** D. Platano, R. Tedeschi, G. Tonini, S. Capone, M. Morri, A. O. Magli, D. Raffa, M. G. Benedetti

**Affiliations:** 1 Physical Medicine and Rehabilitation Unit, IRCCS Istituto Ortopedico Rizzoli, Bologna, Italy; 2 Department of Biomedical and Neuromotor Sciences, Alma Mater Studiorum, University of Bologna, Bologna, Italy; 3 Directorate of the Nursing, Technical and Rehabilitation Service, IRCCS Istituto Ortopedico Rizzoli, Bologna, Italy

**Keywords:** Continuity of patient care, Physical therapy, Rehabilitation, Hip fractures, Elderly

## Abstract

Continuity of care has been linked to patient satisfaction and self-reported outcomes. Following hip fractures in the elderly, rehabilitation aims at restoring patients’ mobility and independence at the pre-fracture level and at the earliest possible time. Despite the potential role of physiotherapists’ continuity on functional outcomes, this correlation has not yet been studied in an acute orthopaedic setting. Guaranteeing the presence of the same physical therapist on individual patients is challenging from an organizational point of view. An observational retrospective study was conducted on 129 aged patients (84 ± 8 years) who underwent surgery for proximal hip fracture. Indicators of outcomes were ILOA score at discharge, length of stay and achievement of rehabilitation goals as defined by the Individual Rehabilitation Project. The number of physical therapists taking care of patients was monitored during the patient’s hospital stay. No correlation was found between the number of physical therapists and functional goals at discharge. The frequent change of physical therapists providing rehabilitation to elderly patients who underwent surgery for hip fragile fracture is not related to functional outcomes.

## Introduction

Policymakers and healthcare providers increasingly express concerns about the fragmentation of care [1]. The literature suggests that increased provider continuity results in higher patient outcomes and satisfaction and lower hospitalization rates [2–4], and that a strong provider–patient relationship is a key factors for relational continuity [5].

In the case of interventions in rehabilitation, especially in frail patients, the concept of continuity includes many aspects of care, and the real discriminating factor is the quality and not the quantity (i.e. longer session) of the rehabilitative intervention [6]. Indeed, it has been reported that the patient–therapist interaction may be more important to the patient than the amount or content of their physiotherapy [7].

In addition, relational continuity is appreciated by patients in terms of satisfaction [8] and might be a motivational driving force for treatment in outpatient setting care. The literature’s data on the correlation between relational continuity and functional outcomes are inconsistent. In post-acute outpatients, Medina and colleagues did not show a correlation between relational continuity and self-reported functional changes [9]. On the other hand, in the home-based setting Russell et al. demonstrated a relationship between the level of continuity in the provider of physical therapy services and the outcomes [10].

Despite the relevance of the topics, few empirical studies have been conducted on the continuity of rehabilitation care, particularly in *acute care*. In this regard, a recent study proved the relationship between the continuity of physical therapy providers and the functional improvement of neurologically impaired inpatients [11]. However, to date, no known studies have studied this relationship in the inpatient orthopaedic population.

In an orthopaedic inpatient acute setting, the short-term functional outcome is a relevant aspect of care and affects discharge time. According to International Guidelines, the rehabilitation process after hip fractures in the elderly should be organized on a multidisciplinary basis; and functional targets are the early restoration of patient mobility and independence at the pre-fracture level (AAOS guidelines, 2021) [12]. To our knowledge, no studies, to date, have studied the relationship between the number of physiotherapists taking care of the patients during their hospital stay and patient’s outcome in hip fragility fractures in an acute inpatient setting. The present study aimed to evaluate whether the number of physiotherapists reflects on short-term functional outcome (evaluated using ILOA score) at discharge in a cohort of aged patients admitted in orthopaedic guards for hip fracture surgery.

## Materials and methods

The study is a secondary analysis conducted on a subgroup of patients enrolled in a previous study [13] that was approved by the Institute’s Ethics Committee CE AVEC: 27/2021/OSS/IOR.

### Study design and participants

A retrospective observational was conducted on 129 aged patients who sustained a proximal femoral fracture and were admitted to various orthopaedic guards between March 2020 and June 2020. Eligibility criteria included patients aged ≥ 65 years who had sustained a proximal hip fracture, and underwent either femur osteosynthesis or hip arthroplasty (total or hemiarthroplasty). Patients with hip fractures due to metastasis or displaying periprosthetic fractures were excluded.

*Rehabilitation programme*: The rehabilitation programme started the day after the surgery. Provided there were medical complications preventing it. Patients participated in two 30-min physical rehabilitation sessions daily during their stay.

*Outcome measures and data collection***:** Data collected included:

- *Demographics*: age, sex
- *Clinical details*: type of hip fracture, treatment method, time to surgery
- *Rehabilitation metrics*: number of sessions, number of physical therapists (PTs) involved per patient and specific PT involvement within the first five days post-surgery
- *Functional assessments*: short-term goals, ASA grade, SAHFE score, ILOA score

The short-term functional objectives were defined by the achievement of the following: (1) sitting with legs out of bed, (2) bed transfer (static and return), (3) chair/wheelchair transfers, (4) walking with a walker, (5) deambulation with crutches and 6—climbing stairs, according to the mobility before admission and the clinical condition of the patient. Other clinical and functional assessments were scored using the following scales: ASA grade of co-morbidities [14], Standard Audit of Hip Fracture in Europe (SAHFE) score (ambulation capacity assessed at admission) and Iowa Level of Assistance Scale (ILOA) score for functional evaluation [15]. ASA scores were assigned: (1) an average healthy patient; (2) a patient with mild systemic disease; (3) a patient with severe systemic disease; (4) a patient with severe systemic disease that is at constant threat to life; (5) a dying patient who is not expected to survive without the operation; and (6) a declared brain-dead patient whose organs are being removed for donor purposes. An ASA score < 3 indicates healthy people or people with mild systemic disease without substantive functional limitations. SAHFE scores were: (1) independent outdoor ambulation; (2) Outdoor ambulation with aid; (3) indoor ambulation with aid (except walker); (4) indoor ambulation with a walker; and (5) unable to walk/using a wheelchair [16]. ILOA score is a 5 functional activity test and gait speed; the score of the first four tasks is based on the level of help needed by the patient for the safe execution of the activity and it ranges from 0 = independent to 6 = not tested for safety reasons; the gait speed is rated according to the time taken to cover a distance of 13.4 m, ranging from 0 (≤ 20 s) to 6 (≥ 70 s). Higher scores represent more assistance and, therefore, more disability. ILOA score was evaluated five days after the beginning of the physiotherapy or at hospital discharge whenever earlier (real functional outcome, score 0–50).

## Data analysis

Analysis of covariance (ANOVA) was performed on samples. Pearson’s correlation was performed to evaluate the linear correlation between two data sets. The statistical significance level for all analyses was set at α = 0.05, ensuring robust and accurate inference. Advanced statistical software packages (SPSS Statistics) were utilized to conduct the studies, ensuring meticulous data handling and adherence to scientific standards. For all tests, *p* < 0.05 was considered significant. Statistical analysis was performed using the Statistical Package for the Social Sciences (SPSS) software version 15.0 (SPSS Inc., Chicago, USA).

## Results

Demographic data and clinical characteristics of patients are shown in Table 1.

**Table 1:** Demographic and clinical characteristics of study sample

Variable	*n *(%)
Sex	
Male (*n* = 23)	23 (17.8)
Female (*n* = 106)	106 (82.2)
Type of surgery	
Plate and screws	71 (55)
Prosthesis	58 (45)

Variable	Mean (SD)
Age (*y*)	84 (8)
ASA	2.8 (0.5)
SAHFE^$^	1.6 (1.0)
ILOA at discharge	38.8 (6.8)

$*n* = 102

Short-term functional goals, as defined by the Physiatric Individual Rehabilitation Project, were achieved in the majority of patients (*n* = 116, 89.9%). Short-term functional goals are shown in Table 2.

**Table 2:** Functional goals as defined by the Physiatric Individual Rehabilitation Project

Functional goals	*n *(%)
Sitting in bed with legs out	7 (5.4)
Chair/wheelchair transfers	8 (6.2)
Bed transfer (static and return)	9 (7.0)
Walking with a walker	82 (63.6)
Deambulation with antibrachials	10 (7.8)
Stairs climbing	13 (10)

Recovery metrics are shown in Table 3.

**Table 3:** Functional goals

	Mean ± SD	Variance	95% CI lower	95% CI upper
Time of achievement of short-term rehabilitation goals (day)	5.32 ± 2.68	7.18	4.85	5.79
Length of stay (day)	10.9 ± 4.19	17.6	10.17	11.63
Length of stay before surgery (day)	1.49 ± 1	0.99	1.32	1.66
Length of hospital stay after surgery (day)	9.38 ± 4.12	16.9	8.66	10.10
Physiotherapy starting day	1.33 ± 0.79	0.63	1.19	1.47
Total number of physical therapists per patient	5.72 ± 2.85	8.14	5.22	6.22

We found that patients who did not achieve the rehabilitation objectives (*n* = 13, 10.1%) had statistically significant longer preoperative stay (2.00 days ± 0.34SD vs 1.43 days ± 0.088SD, *p* = 0.041) and higher ILOA score (42.46 ± 1.43SD vs 38.41 ± 0.64SD, p = 0.016) at discharge when compared to patients who reached their goals. As expected, we found a correlation between age and ILOA at discharge (*r* = 0.41, *p* < 0.001).

The time of achievement of short-term rehabilitation goals was five days (i.e. 5.32 ± 2.68 days), while the length of stay after surgery was l9.38 ± 4.12 days.

We found that ambulation with crutches was achieved later and by a significantly lower percentage of patients and that most patients (81.4%) could ambulate with aids. The Cohen’s d effect size is summarized in Table 4. The smallest effect size (0.14) was found for the total number of PTs per patient in relation to the time necessary to achieve the rehabilitative goal.

**Table 4:** Summarizing the Cohen’s d effect sizes

	Time of achievement (days)	LoS (days)	LoS before surgery (days)	LoS after surgery (days)	Physiotherapy starting day	Total n of PTs per patient
Time of achievement of short-term rehabilitation goals	–	1.59	1.89	1.17	2.02	0.14
LoS	1.59	–	3.09	0.37	3.17	1.45
LoS before surgery	1.89	3.09	–	2.63	0.18	1.98
LoS after surgery	1.17	0.37	2.63	–	2.71	1.03
Physiotherapy starting day	2.02	3.17	0.18	2.71	–	2.10
Total n of PTs per patient	0.14	1.45	1.98	1.03	2.10	–

We evaluated the correlation between the number of physical therapists who treated a patient within five days and the functional score, and we found an inverse correlation between the number of PTs and the ILOA score (Rho = − 0.189 *p* = 0.032). No correlation was found between number of physical therapy providers and functional goals at discharge, in terms of both ILOA scores (*R*^2^ = 0.0004, *r* = 0.0019) and rehabilitation objectives (*R*^2^ = 0.0059, *r* = − 0.077), when the entire length of stay in the hospital after surgery was considered.

## Discussion

To our knowledge, this is the first study to investigate the relationship between the number of physical therapists treating geriatric patient and the functional outcome in an acute orthopaedic setting. Our data do not show a significant correlation between continuity of physiotherapists and functional outcomes, and they differ from what was recently reported by Adam et al., 2023 [11] on inpatient neurological patients. The reason for different results may rely on several factors. Namely, the *different diagnoses* (i.e. neurological vs orthopaedic) and subsequent disability which lead to the higher dependency of patients on their therapists since neurological patients might be limited not only in their mobility but also in other functions such as communication ability, nutrition and grooming. Indeed, Adam and colleagues found the highest inverse correlation between the number of PT and functional outcomes in the subgroup of “stroke,” “spinal cord injury” and “other neuromuscular” patients. The *intensity* of rehabilitation may be another reason since typically inpatient rehabilitation consists of at least 3 h of therapy per day, while in the present study, patients underwent two 30-min sessions of physiotherapy per day. Another aspect to consider is the *age of patients*, which was higher in our study (84 ± 8 vs 60.8 ± 15.9 years in the study by Adam et al., 2023). Ageing, combined with *frailty* (a well-described age-dependent reduction of patients’ multisystem physiological and psychological reserve), may impact the medical conditions in the postoperative period [17], increasing the risk of adverse outcomes, especially mortality and delirium, with delays or interruptions of the functional recovery, resulting in a prolonged hospital stay [18, 19].

Finally, since the retrospective study was conducted from January to March 2020, the simultaneous outset of the COVID-19 pandemic introduced additional variables and exacerbated typical stressors within therapeutic settings, amplifying practitioner anxiety and increasing patient emotional frailty due to restricted visitation policies [20]. Some other biases may have influenced the cohort of patients. Specifically, in the studied population, the SAHFE score was 1.6 ± 1, indicating a moderate–high pre-recovery functional level despite the average age of 84 ± 8 years.

The “Iowa Level of Assistance” (ILOA) was used to assess functional recovery and the level of independence at discharge, and it is a tool adequate to evaluate the outcome of the early rehabilitation treatment; we found that it correlated with age (*r* = 0.41, *p* < 0.001) and that this observation is in accordance with the previous literature [21].

In the present study, the total number of physical therapists per patient was 5.72 ± 2.85SD, and the average length of stay in the hospital after surgery was 9.38 ± 4.12 days, indicating a high level of therapist rotation, which was not related to standard working shift only. In fact, at the organizational level, the continuity in rehabilitation care is a result of balancing the needs of both patients and employees [22]: Sick leave, employee holidays, turnover, other types of absences and rotations in different hospital wards definitively affect the continuity of care. Likely, the COVID pandemic’s first phase of impact on sanitary personnel may have contributed to a further increase in care fragmentation. This topic could be further explored through additional research.

The present retrospective study has several limitations: It focuses on very short-term outcomes and lacks insights into the long-term efficacy of the surgical and rehabilitative interventions. The absence of data about the preoperative functional ability of the patients minimizes the depth of functional outcome analyses. Additionally, being conducted within a singular, specialized orthopaedic hospital, the generalizability of the findings to broader populations and disparate clinical settings is potentially limited.

Further studies are needed to explore the role of PTs in inpatient orthopaedic rehabilitation on other age groups, and especially on long-term functional outcomes.

## Conclusions

The present data provide evidence that, in an acute orthopaedic inpatient setting, the short-term functional outcomes depend on the precocity of the treatment (management continuity) rather than on relational aspects of the continuity of care of a single physical therapist.
